# Anorexia, Capacity, and Best Interests: Developments in the Court of Protection Since the Mental Capacity Act 2005

**DOI:** 10.1093/medlaw/fww037

**Published:** 2016-12-22

**Authors:** Beverley Clough

**Affiliations:** 1.24, Liberty Building, School of Law, University of Leeds, LS2 9JT

**Keywords:** Anorexia nervosa, best interests, medical experts, mental capacity, therapeutic jurisprudence, UNCRPD

## INTRODUCTION

Anorexia nervosa has long been the subject of medical and psychiatric debate, yet it is only relatively recently that English law has been called upon to grapple with the complexity of this condition. It was not until the early 1990s that we saw English law’s involvement with anorexia nervosa and questions as to the legitimate powers of the medical profession regarding involuntary treatment.^1^ The most recent Court of Protection decision in *An NHS Foundation Trust v Ms X*^2^ invites critical reflection on the impact that the Mental Capacity Act 2005 (hereafter the MCA) has had on the legal and medical discourse surrounding cases involving individuals with anorexia nervosa. This clinically and ethically complex case brings into sharp focus the tensions pervading the use of the Mental Capacity Act 2005^3^ to compel medical treatment in the context of anorexia. It is salient to consider the case in light of the previous cases involving individuals with anorexia, *Re L*^4^ and *Re E*,^5^ and reflect on what we can discern from the judgment and reasoning going forward. Keywood, in considering the earlier decisions regarding force-feeding of women and minors with anorexia nervosa, suggested that English law adopted a ‘crude, biomedical explanation’ of the condition, and that the application of the (then nascent) threshold of incapacity was ‘controversial and problematic’.^6^ Conversely, one of the central claims that heralded the MCA, and which regularly appears in discussions of the MCA in academic and policy literature, is the idea that the legislation is ‘empowering’ for those with disabilities.^7^ What impact has the MCA had in relation to patients with anorexia, in terms of framing and responding to the ethical and medico-legal questions raised in this context, and does the developing jurisprudence in this context demonstrate an increasingly empowering, humane, and attentive legal framework? The commentary will consider whether the developments in the case law post-MCA represent an important turning-point in how the law responds to patients with anorexia, and ‘best interests’ more generally. This is particularly salient when considered in light of the increasing attention being paid to the UN Convention on the Rights of Persons with Disabilities (UNCRPD) and the emphasis on the will and preferences of the individual as being paramount. An important alternative interpretation of the case law in this context is further raised, noting the need to reflect on whether the law is still deferential to medical expert opinion as a guiding consideration. Increasing attention is being focused on veiled sources of influence on the development of medical law, and Montgomery, Jones and Biggs have recently highlighted the hidden law-making in medical jurisprudence.^8^ They have drawn attention to the way in which the development of law in ethically contentious areas such as medical law is subject to diverse and often hidden influences from sources which are not typically recognized in traditional debates about the separation of powers and legitimate role of the courts. These observations resonate with the concerns highlighted in this article and warrant further reflection. As will be discussed, the question of resources can also be seen to play an important role in this trio of cases—a role which should not be overlooked if arguments for facilitating rights for people with cognitive impairments are to be endorsed in the Court of Protection.

## THE FACTS OF THE CASES^9^

The first case to face the Court of Protection following the MCA, *Re E*, involved a 32-year old woman with a history of severe anorexia nervosa in addition to alcoholism and a personality disorder. Described by Jackson J. as ‘an intelligent and articulate woman’,^10^ E had spent a considerable amount of her adult life receiving treatment for anorexia with little success, and had attempted to make an advance decision refusing treatment. She had ‘pleaded’ with Dr Glover, the medical expert in the case, not to make her endure further treatment for her anorexia. The opinion of E’s parents, and her treating medical team, was that E lacked capacity in relation to nutrition and interventions such as force-feeding, but that it was not in her best interests to be force-fed. They placed great emphasis on the long period of time which E had compulsorily been treated in the past, without success, and argued that she should now be given the right to ‘choose her own pathway’.^11^ All things considered, however, Jackson J declared E lacked mental capacity and that it was in E’s best interests to receive further treatment for her anorexia nervosa against her wishes, due in part to the views of the court appointed expert, Dr Tyrone Glover, that a new, previously untried treatment may be successful.^12^

Later that year, in *Re L*, King J was faced with a 29-year old woman with a long history of anorexia nervosa who had spent around 90% of her life over the previous 16 years as an inpatient in various units. At the time of the case, L weighed around three stone and had a very poor prognosis. Again, it was seen as uncontroversial in the case that L lacked capacity to make decisions in relation to serious medical treatment, including in relation to nutrition and hydration. The question arose as to whether it is in L’s best interests to forcibly re-feed her. L’s family and medical team were all agreed that invasive force-feeding was not in her best interests, given the length of time that L had suffered from anorexia nervosa. The medical evidence (provided by Dr Glover) was that the act of inserting a naso-gastric or PEG tube, and the sedation to do this, would lead to almost certain death^13^ due to her frail physical condition and severely impaired organ function. As such, it was held that force-feeding was not in L’s best interests and thus that it was lawful to withhold such treatment.

The most recent case, *Ms X*, involves a young woman suffering from a complex interaction of severe anorexia nervosa and alcohol dependence syndrome. Her anorexia has dominated her life for the previous 14 years and has been compounded by her alcoholism, believed to be fuelled by harmful childhood experiences. This had resulted in hospital admissions for acute renal failure and multiple-organ failure which at one point had caused her to descend into a coma. She had also had over 45 admissions to hospital for her anorexia, sometimes receiving treatment under the Mental Health Act 1983. In a damaging cycle, whenever Ms X has been treated as an in-patient at specialist units and has been force-fed to reverse the effects of her anorexia, she has binged excessively on alcohol upon discharge, purportedly to blunt her distress.

Typically, the purpose of re-feeding a patient with anorexia is to keep them alive while talking therapies such as psychotherapy are facilitated to try to treat the underlying anorexia. This was incredibly difficult in this case, however, as Ms X had not responded well to talking therapies and had refused to engage in counselling. She was reported in the evidence of one of the medical experts, Dr Tyrone Glover, as saying that ‘I don’t like talking about these things. I don’t do talking. I have never opened up to therapists and I never will. I don’t like it’.^14^ This led Dr Glover to conclude that ‘no one can be coerced into psychotherapy and so this avenue is closed and most likely the time for such treatment has long since passed’.^15^ Against this complex background of comorbidities and repeated unsuccessful admissions for treatment, the Court of Protection held that Ms X lacked capacity to make decisions about her treatment in relation to anorexia, and that it would not be in her best interests to force-fed her against her will.

## DISCUSSION

### A. Capacity

One of the striking aspects of this trio of cases is the way in which the individual in each case is consistently found to lack mental capacity under the MCA. In *Re E,* Jackson J stated that, in relation to capacity:

… there is strong evidence that E's obsessive fear of weight gain makes her incapable of weighing the advantages and disadvantages of eating in any meaningful way. For E, the compulsion to prevent calories entering her system has become the card that trumps all others. The need not to gain weight overpowers all other thoughts. By way of example, in August 2011, she was described as smiling and laughing during a conversation, but when the question of weight gain and the achievement of a BMI of 16 was mentioned, she began to cry.^16^

Similarly, in *Re L,* King J outlined how L showed ‘inappropriate indifference to matters of life and death and it seems as if it has not entirely hit home’,^17^ and that her profound fear of weight gain prevented her from being able to weigh up the risks and benefits of medical treatment. Finally, in *Ms X,* Evidence was given by Dr A that in relation to her understanding of the issues around her anorexia nervosa:

Ms X is able to understand the information provided and on my assessment of her cognitive state on the 28th August 2014 she was able to retain and feedback to me the information provided to her about the same evidencing both retention and understanding. However due to ongoing severe body dysmorphia, false beliefs about her weight shape and nutritional state and absolute fear of weight gain from her anorexia, she was and is unable to apply the information to herself or believe in the need for it. The reality and importance of the associated risks including death of her malnourished state are therefore not truly appraised which means she is unable to weigh up the information provided in the decision making process.^18^

Dr A and Dr Glover both agreed that Ms X lacked capacity due to her inability to weigh the relevant information. Based on these assessments, Cobb J. was untroubled in concluding that Ms X lacked capacity to make decisions about her eating disorder.

While it could be said that the fact that all three cases involved a finding of incapacity may simply be due to the facts of each particular case, it is a trend which has pervaded all of the anorexia cases which have come before the courts, including those prior to the MCA. Indeed, E’s parents recognized this salient point, saying:

It seems strange to us that the only people who don't seem to have the right to die when there is no further appropriate treatment available are those with an eating disorder. This is based on the assumption that they can never have capacity around any issues connected to food. There is a logic to this, but not from the perspective of the sufferer who is not extended the same rights as any other person.^19^

It appears from these cases that there is essentially an ‘absolute presumption’^20^ that an anorexia nervosa patient lacks the capacity to refuse interventions such as direct feeding for their eating disorder. It seems that in this respect, the post-MCA case law has shown little by way of development of legal understanding of the complexity of anorexia and its impact on the abilities of those with the condition. It might here be argued, as Wang has, that the way in which those with anorexia are in practice presumed to lack capacity to make decisions about treatment for their eating disorder demonstrates that the application of the MCA here is incompatible with the UNCRPD^21^ (discussed in more detail below). He sees this as a denial of the right to make an autonomous decision on where the balance lies between the quality and duration of their life.^22^

It is important to reflect further here on the consistent finding of incapacity in relation to the person with anorexia in light of the way in which the test and threshold for capacity was developed. While it appears that the test for capacity in the MCA, stemming as it did from *Re C*,^23^ is a legal construct, its origins belie a more than subtle medical influence. As Keywood explains, in the case of *Re C,* the medical expert Dr Nigel Eastman was invited to give his assessment of the capacity of Mr C. His assessment drew on three factors which he saw as relevant to assessing capacity in relation to health care decisions, namely retaining information; believing information; and weighing up that information.^24^ Keywood highlights how Thorpe J then ‘borrowed’ these criteria to form the legal threshold of mental capacity.^25^ Reflecting upon this conceptualization of capacity, heavily rooted in clinical thinking, allows us to see the way in which the courts have struggled to fully grasp the full complexity of anorexia. Keywood argues that ‘this judicial annexation of a clinical assessment measure is highly problematic for it conceals the fact that clinical determinations of patients’ decision-making abilities are given a normative force that they were never intended to have. Moreover, the manner of this judicial appropriation has meant that a range of nonclinical issues that impact on people’s decision-making abilities have been obscured from critical examination by the courts’.^26^ Instead, the narrow, biomedical view which can be traced through all of the anorexia case law has survived the passing of the MCA. Significantly, in this context more than others, there is an enduring insensitivity to the social and political context of anorexia, and a persistence of value judgements about the agency of the person with anorexia.

### B. Best Interests

One of the aspects of *Ms X* which has been most welcomed is the way in which her best interests were considered. Cobb J. began his discussion of best interests with a nod towards the law’s granting of the ‘highest (even if not absolute) priority to the preservation and sanctity of life’,^27^ pointing to the way in which this corresponds with common law obligations, Article 2 of the European Convention on Human Rights,^28^ s 4(5) of the MCA,^29^ and the MCA Code of Practice.^30^ Indeed, he stated that Ms X ‘retains an interest in life, and has plans for her future- including ‘visiting places’, spending time with her beloved grandfather, distance learning and enjoying music’.^31^ Notwithstanding her wishes not to be compelled to receive treatment, she has no wish to die.

In ascertaining Ms X’s best interests, taking the above into account, Cobb J. framed the relevant question in light of *Aintree University NHS Foundation Trust v James*^32^—the first time in which the Supreme Court had applied the MCA—in which Lady Hale stated:

… the focus is on whether it is in the patient’s best interests to give the treatment, rather than on whether it is in his best interests to withhold or withdraw it. If the treatment is not in his best interests, the court will not be able to give its consent on his behalf and it will follow that it will be lawful to withhold or withdraw it. Indeed, it will follow that it will not be lawful to give it.^33^

Furthermore, Lady Hale emphasized the way in which an appraisal of best interests in a given case should be undertaken. In an important passage, she stated that:

[I]n considering the best interests of this particular patient and at this particular time, decision-makers must look at his welfare in the widest sense, not just medical but social and psychological; they must consider the nature of the medical treatment in question, what it involves and its prospects of success; they must consider what the outcome of that treatment for the patient is likely to be; they must try and put themselves in the place of the individual patient and ask what his attitude to the treatment is or would be likely to be.^34^

Applying this to Ms X and the issue of re-feeding, Cobb J. was mindful of the fact that this would impose a considerable restriction on her liberty and reduce her quality of life considerably by removing her from close friends and family.^35^ Moreover, evidence from Ms X’s records suggest a high probability of suicide attempts or other self-harming behaviours^36^ if she were to be forcibly re-fed, particularly given that recourse to relief through alcohol use would be cut off. The distress and trauma associated with the insertion of the naso-gastric tube and restraint involved in this process, and the potential complications involved in this due to the presence of varicose veins in Ms X’s throat (caused by the combination of liver disease and previous naso-gastric feeding treatments) further persuade against the compulsion of medical treatment here.^37^ In terms of Ms X’s wishes and feelings,^38^ the medical experts have elicited her views and recount them in the case,

Ms X fully agrees with [the application] and has repeatedly requested that we do not detain or forcibly feed her. She is very clear that in her experience the use of the MHA to enforce nutritional treatment serves only to make the situation and experience worse for her, not better. Ms X feels that not having the continued threat of MHA detention and treatment will also allow her control and the ability to make decisions about her care plan.^39^

More importantly, Ms X herself sent a letter to the Official Solicitor on the eve of the hearing, stating her views on the application and her treatment. She stated, among other things that

I understand the professionals concerns and the effect that this has had on all of them and I do recognise that everyone wants for the best. However … rather than helping me, it is actually making me worse … but I am also fully aware that there is support and treatment still available if I ever want it.^40^

As noted above, the complex interplay of conditions in *Ms X* makes the legal issues fraught with difficulty. This is nowhere more stark than in the ‘paradox’ that is central to the best interests decision facing Cobb J. The concern, echoing the medical evidence and past behaviour, is that if feeding were to be compelled, the court may be ‘facilitating or accelerating the termination of her life’.^41^ Yet this tension reaches even deeper, as there is evidence to suggest that if Ms X ‘retains her autonomy’^42^ then she may access some medical help, even if just of a palliative nature. Evidence suggests that she may not be seeking palliative help for the physical symptoms of her liver disease because she is concerned that this may result in her detention to hospital for forced-feeding.

It is salient to note here the importance of the individual’s voice being heard through Court of Protection cases. Until recently, it was rare for the Court of Protection judge to visit the individual whose capacity was in question. What is striking, however, is the impact that the involvement of the individual can have on the outcome of the case. As cases such as *CC v KK and STCC*,^43^ and more recently *Wye Valley v B*^44^ demonstrate, the involvement of the individual can serve as a vital reminder of the human aspects of the decision facing the court—an aspect that can all too easily be forgotten in the legal process.

In this regard, *Ms X* is of note as an example of a particularly humane and carefully considered decision as to best interests. Cobb J. sought to apply the familiar legal framework with a constant eye towards the complex interweaving of conditions, and with laudable consideration of Ms X’s wishes. The focus on the probable consequences of his assessment was also clear throughout, with emphasis on the way in which enforcing treatment may remove the opportunity for engagement with palliative care.^45^ This added an interesting therapeutic jurisprudential angle to the case, which emphasizes the way in which a judgement can aid the psychological and clinical outcomes for the individual.^46^ The literature on therapeutic jurisprudence has emphasized the importance of using the law to empower people and enhance rights, and has been described as ‘… a sea change in ethical thinking about the role of law … a movement towards a more distinctly relational approach to the practice of law … which emphasizes psychological wellness over adversarial triumphalism’.^47^ Such an approach is in many ways a reflection of a more grounded and humane approach to best interests which is aligned with seeing people deemed to lack mental capacity as ‘subjects rather than objects’.^48^

How can this be reconciled with the other post-MCA cases? Does the decision in *Ms X* signal a swing towards a more nuanced understanding of anorexia? *Re E* was criticized for failing to respect E’s strongly held wishes and forcing her to endure unwanted and possibly undignified treatment.^49^ Indeed, we caught a rare glimpse of the post-script to E’s case in *Ms X*, as we were told that at the time of the judgement, E was still receiving inpatient care. In some ways, the decision in *Ms X* may be lauded as demonstrating the respect which ought to be granted to the wishes of the individual deemed to lack capacity. The emphasis placed on Lady Hale’s statements in *Aintree v James* by Cobb J. is telling in this regard and highlights the importance of seeing things subjectively from the patient’s perspective. This development similarly accords with burgeoning debates on the compatibility of the MCA with the UNCRPD. The UNCRPD can be seen as a key turning-point in the recognition of the rights of individuals with disabilities, and those with psycho-social disabilities are expressly brought within the remit of the Convention.^50^ The UNCRPD places an emphasis on positive obligations on states to ensure that rights are secured to people with disabilities and that substantive equality is secured. The UK has signed and ratified the Convention and while it is not incorporated within English law it may be drawn on as an interpretative aid.^51^ In relation to the MCA, proponents of the UNCRPD emphasize that Article 12 of the Convention casts doubt on the compatibility of the current legal framework.^52^ Article 12 requires ‘equal recognition before the law’ for people with disabilities and stresses that this equal recognition should be enjoyed by all. In a long-awaited General Comment on Article 12, the Committee were keen to stress that the conflation of legal capacity (composed of legal standing and legal agency) with mental capacity (judgments about decision making skills) which has been used to justify systems of substitute-decision making or guardianship are to be abolished under the CRPD.^53^ Instead of relying on such an approach, the Committee stressed the need to provide support to exercise legal capacity, including supported decision making. A key aspect of this is the focus on eliciting the will and preferences of the individual,^54^ and that these ought to be respected. Doubt is thus cast on the compliance of the MCA with these international obligations, and the review of the UK’s compliance with the UNCRPD—which is being undertaken imminently—will be awaited with interest. Viewing *Ms X* in light of these discursive developments, however, suggests that greater recognition is being given to the primacy of the wishes of the individual, and this is a welcome trend for many with an interest in disability rights.

However, in analysing these cases side-by-side, it is also evident that the courts are not always necessarily engaging closely with the will and preferences of the individuals involved. While on one level, it seems that (in *Re L and Ms X* at least) the wish not to be forcibly fed is being respected by the courts, this is not necessarily a true reflection of the complexity of views. In all of the cases, the women stated clearly that they did not wish to die.^55^ In *Re L,* there is some brief discussion of L’s desire to move to a nursing home.^56^ It transpired that she had been due to move to one previously, but the home withdrew its offer of a bed, to which L reacted by reducing her food intake and becoming dangerously ill again.^57^ Later on in the judgment, mention is made of L’s desire to stay alive and her hope of becoming strong enough to move to a nursing home. Further written evidence stated that L felt that if a nursing home place was secured and funding put in place, she would have the motivation to move forward.^58^ With this in mind, it is somewhat disappointing that this is not closely engaged with by King J, and it can only be said with hesitation that L’s will and preferences were being addressed here. Closer engagement with the complexity and nuances of a persons’ wishes in these judgments would go some way to ensuring that ‘will and preferences’ or ‘beliefs and values’ are not just addressed in a tokenistic and simplistic manner.

Further analysis of these cases provides an alternative light in which the decisions can be viewed; one which does not so readily suggest a ‘triumph’ for patient autonomy.^59^ Reading the decisions more closely, it is instructive to note that the outcome in each case was also the outcome which the expert opinion tended to favour. While of course the cases can be reconciled simply by pointing to the fact that cases should be dealt with on their own facts, and any ‘quirk’ in the outcomes can simply be put down to individual differences between them, it is worth considering the dominance of medical expertise in the court room and the extent to which this, if ever, is challenged by the judges in relation to capacity and best interests. As noted above, in *Re L*, for example, the expert opinion stated that if L were to be forcibly fed by naso-gastric tube, there was an almost 100% likelihood of death and subsequently it was held that it was not in L’s best interests to be forcibly re-fed. In *Re E,* the impact of expert opinion is more starkly seen. In this case, there was some disagreement between the experts, some of whom had worked with E in the past, as to the likelihood of success of future re-feeding. The doctors who had been treating E were doubtful about further coercive treatment, feeling that all avenues had been considered and E was strongly against such treatment. Notably the staff, while doubtful about future treatment, were willing to accept whichever course the judge decided. The Health Authority retained a neutral stance and did not launch any positive case against forcible-feeding. The medical expert, Dr Glover, however, opined that treatment which might return E to a relatively normal life is available and has not thus far been tried.^60^ Crucially, we see that a bed is available at a specialist unit and that the Health Authority was willing to pay for it.^61^ Indeed, Jackson J, in declaring it to be in E’s best interests to undergo this treatment, stated that:

I record that the state, having instigated this plan of action for E in the way that it has, is now honour bound to see it through by the provision of resources in the short, medium and long term. Had the authorities not made that commitment, I would not have reached the conclusion that I have.^62^

This is a very strong and telling statement by the judge, and illustrates the presence of professional medical opinion and resources which are often the unseen undercurrents in such cases, which facially purport to be about the ‘big’ ethical questions of autonomy and self-determination. In the present case of *Ms X,* it is seen that there is no medical opposition to the outcome which is contended for.^63^ While it is not being argued that the outcome of these cases is wrong, or that the law was not applied correctly, it is salient to note the sway that medical opinion and resources can have on the decision. Anorexia nervosa has a complex and contested status and while the cases in the Court of Protection arise at a crisis point, focus must also be directed to the surrounding societal context, including access to services, in order to convincingly argue that we are upholding the rights of the person. Similarly, while the therapeutic jurisprudential angle of *Ms X* is to be welcomed, and may represent moves towards a more nuanced response to the individual, it is still problematic that incapacity is almost presumed in cases involving an individual with anorexia. This signals the retention of medico-legal control over the subject, and the subjection of the patient’s best interests to this gaze.

## Footnotes

1 See *Re W (a minor) (medical treatment)* [1992] 4 All ER 627; *Re KB (adult) (mental patient: medical treatment)* (1994) 19 BMLR 144; *F v Riverside Health Trust* (1994) 20 BMLR 1.

2 *A NHS Foundation Trust v Ms X* [2014] EWCOP 35.

3 Hereafter referred to as the MCA.

4 *The NHS Trust v L* [2012] EWHC 2741 (COP).

5 *Re E (Medical treatment: Anorexia)* [2012] EWCOP 1639.

6 K Keywood, ‘Rethinking the Anorexic Body: How English Law and Psychiatry “think”’ (2003) 26 Int J L Psychiatry 599–616, 601.

7 See, for example, Lord Falconer’s statement in the foreword to the Code of Practice; ‘it will empower people to make decisions for themselves wherever possible’. Department for Constitutional Affairs, *Mental Capacity Act Code of Practice* (TSO 2007).

8 J Montgomery, C Jones and H Biggs, ‘Hidden Law-Making in the Province of Medical Jurisprudence’ (2014) 77(3) Modern L Rev 343–78.

9 For a more detailed outline of the facts of *Ms X* and *Re E,* see John Coggon’s previous commentaries in this journal: J Coggon, ‘Alcohol Dependence and Anorexia Nervosa: Individual Autonomy and the Jurisdiction of the Court of Protection’ (2015) 23(4) Med L Rev 659–67; J Coggon, ‘Anorexia Nervosa, Best Interests and the Patients Human Right to a “Wholesale Overwhelming of her Autonomy”’ (2014) 22(1) Med L Rev 119–30.

10 *Re E* [2012] EWHC 1639 (COP) para 75.

11 Ibid, para 80.

12 Ibid, para 138.

13 *Re L* (2012) EWHC 2741 (COP) para 44.

14 *Ms X,* para 15.

15 ibid.

16 *Re E*, para 49.

17 *Re L*, para 49.

18 *Ms X*, para 27, emphasis added.

19 *Re E*, para 52.

20 D Wang, ‘Mental Capacity Act, Anorexia, and the Choice Between Life-Prolonging Treatment and Palliative Care: A NHS Foundation Trust v Ms X’ (2015) 78(5) Modern L Rev p871.

21 ibid p882.

22 ibid.

23 *Re C (Adult: Refusal of Medical Treatment)* [1994] 1 All ER *819.*

24 K Keywood, ‘Rethinking the Anorexic Body: How English Law and Psychiatry ‘Think’’ (2003) 26 Int J L Psychiatry 599–616.

25 ibid p606.

26 ibid p605.

27 See, for example, *W v M and Others* [2011] EWHC 2443 (Fam).

28 Art 2 (1) ‘Everyone's right to life shall be protected by law. No one shall be deprived of his life intentionally save in the execution of a sentence of a court following his conviction of a crime for which this penalty is provided by law’.

29 ‘Where the determination relates to life-sustaining treatment he must not, in considering whether the treatment is in the best interests of the person concerned, be motivated by a desire to bring about his death.’

30 Mental Capacity Act Code of Practice, paras 5.29–5.36 ‘all reasonable steps which are in a person’s best interests should be taken to prolong their life’ and that there will only be ‘a limited number of cases where treatment is futile, overly burdensome to the patient or where there is no prospect of recovery’.

31 *Ms X*, para 38.

32 [2013] UKSC 67.

33 Ibid, para 22.

34 Para 39.

35 *Ms X*, para 41.

36 Dr A, for example, referred to the ‘phenomenal’ threat of suicide, and ‘the risk of other behaviours, cutting or hanging, would escalate considerably’, para 41.

37 See medical evidence in paras 45 and 47.

38 As per Mental Capacity Act, s 4(6).

39 Dr A, para 48.

40 Ms X, para 51.

41 Para 42, per Cobb J.

42 Para 44.

43 [2012] EWHC 2136 (COP).

44 [2015] EWCOP 60: ‘Lastly, I refer to the principle at s. 4(4) that so far as is reasonably practicable, the person must be permitted and encouraged to participate as fully as possible in any decision affecting him. In this case, given the momentous consequences of the decision either way, I did not feel able to reach a conclusion without meeting Mr B myself. There were two excellent recent reports of discussions with him, but there is no substitute for a face-to-face meeting where the patient would like it to happen. The advantages can be considerable, and proved so in this case. In the first place, I obtained a deeper understanding of Mr B's personality and view of the world, supplementing and illuminating the earlier reports. Secondly, Mr B seemed glad to have the opportunity to get his point of view across. To whatever small degree, the meeting may have helped him to understand something of the process and to make sense of whatever decision was then made. Thirdly, the nurses were pleased that Mr B was going to have the fullest opportunity to get his point across. A case like this is difficult for the nursing staff in particular and I hope that the fact that Mr B has been as fully involved as possible will make it easier for them to care for him at what will undoubtedly be a difficult time.’ Per Jackson J, para 18.

45 *Ms X,* para 44. Also see para 59.

46 D Wexler and B Winick, *Essays in Therapeutic Jurisprudence* (Carolina Academic Press 1992).

47 W Brookbanks, ‘Therapeutic Jurisprudence: Conceiving an Ethical Framework’ (2001) 8 J L Med p328. Also see ML Perlin, ‘“The Ladder of the Law has no Top and no Bottom”: How Therapeutic Jurisprudence can give Life to International Human Rights’ (2014) 37 Int J L Psychiatry 535–42 for a discussion of the need for synergy between therapeutic jurisprudence and international human rights discourse, with particular regard to the UN Convention on the Rights of Persons with Disabilities 2007.

48 G Quinn, T Degener and A Bruce, *Human Rights and Disability: The Current Use and Future Potential of United Nations Human Rights Instruments in the Context of Disability* (United Nations 2002) p9.

49 C Ryan and S Callaghan, ‘Treatment Refusal in Anorexia Nervosa: The Hardest of Cases’ (2014) 11(4) Bioethical Inquiry 43–45; T Hayes, ‘Making Decisions: Balancing Principles, Forcing Food: Self-determination and Best Interests’ (2012) 9(4) Bioethical Inquiry 387–91.

50 See art 1.

51 We have seen the domestic courts gradually begin to grapple with the CRPD in judgments, see for example *AH v West London MHT* [2011] UKUT 74 (AAC) para 16; *A Local Authority v TZ* (No 2) [2014] EWCOP 973.

52 See L Series and others, ‘Mental Capacity Law Discussion Paper-The Mental Capacity Act 2005, the Adults with Incapacity (Scotland) Act 2000 and the Convention on the Rights of Persons with Disabilities: The Basics’ 39 Essex Street Newsletter, June 2014, <http://www.39essex.com/docs/newsletters/crpd_discussion_paper_series_et_al.pdf> accessed 5 September 2016. Also see W Martin and others, Achieving CRPD Compliance: Is The Mental Capacity Act of England and Wales Compatible with The UN Convention on The Rights of Persons with Disabilities? If Not, What Next?, Essex Autonomy Project (2014).

53 Office of the High Commissioner for Human Rights, ‘Committee on the Rights of Persons with Disability General Comment No 1: Article 12 Equal Recognition Before the Law’ (Eleventh Session, 31st March–11th April 2014) paras 13–15.

54 Art 12(4).

55 See N Munro, ‘Taking Wishes and Feelings Seriously: The Views of People Lacking Capacity in Court of Protection Decision-Making’ (2014) 36(1) J Social Welfare Family L 59–75, p66 for a discussion of this.

56 *Re L*, para 23.

57 ibid.

58 Para 31.

59 It is beyond the scope of this commentary to discuss here the nature of autonomy, but see critical literature on autonomy and the MCA such as, J Coggon and J Miola, ‘Autonomy, Liberty, and Medical Decision-Making’ (2011) 70(3) Cambridge L J 523–47; N Munro, ‘Taking Wishes and Feelings Seriously: The Views of People Lacking Capacity in Court of Protection Decision-Making’ (2014) 36(1) J Social Welfare Family L 59–75; F Freyenhagen and T O’Shea, ‘Hidden Substance: Mental Disorder as a Challenge to Normatively Neutral Accounts of Autonomy’ (2013) 9(1) Int J L Context 53–70.

60 *Re E*, para 38.

61 ibid, para 93.

62 Para 143.

63 Para 37.

